# Diabetic vascular hyperpermeability: optical coherence tomography angiography and functional loss assessments of relationships among retinal vasculature changes

**DOI:** 10.1038/s41598-021-83334-6

**Published:** 2021-02-18

**Authors:** Mitsuru Arima, Shintaro Nakao, Yoshihiro Kaizu, Iori Wada, Muneo Yamaguchi, Kohta Fujiwara, Masato Akiyama, Alan W. Stitt, Koh-Hei Sonoda

**Affiliations:** 1 Department of Ophthalmology, Graduate School of Medical Sciences, Kyushu University, Fukuoka, Japan; 2 Department of Ophthalmology, National Kyushu Medical Center, 1-8-1, Jigyo-hama, Chuo-ku, Fukuoka, 8108563 Japan; 3 Department of Ophthalmology, Fukuoka University Chikushi Hospital, Fukuoka, Japan; 4 Centre for Experimental Medicine, Queen’s University Belfast, Belfast, Northern Ireland

**Keywords:** Retinal diseases, Diabetes complications

## Abstract

Our study assessed the influence of vascular permeability on vascular flow density (FD)-correlated retinal sensitivity (RS) in DR. In this cross-sectional, prospective, consecutive study, RS in the extrafoveal macula of DR patient was measured by microperimetry. FD was measured in the total, superficial, and deep capillary plexus layers (TCP, SCP, and DCP) by optical coherence tomography angiography. All measurement points were classified into four categories according to intensity of fluorescein leakage and FD, and the RS reduction was compared. A stratified analysis by retinal thickness (RT) was also performed. Fourteen eyes (14 patients) were enrolled. FDs at 207 RS measurement points were analyzable. For TCP, SCP and DCP, the leakage did not decrease RS at points where FD was maintained. The greater the leakage, the smaller the RS reduction at points with low FD in TCP (*P* = .020). Points with high leakage showed a significant smaller RS reduction than points with low leakage (*P* = .001 for TCP, *P* = .040 for SCP, and *P* = .046 for DCP) only in areas with low RT and low FD. Our results suggested that vascular hyperpermeability may inhibit the RS reduction in the non-edematous ischemic diabetic retina.

## Introduction

Diabetic retinopathy (DR) is a major microvascular complication of diabetes mellitus and a leading cause of vision loss in working-age adults^1,2^. Vascular abnormalities, including hyperpermeability-associated retinal edema and ischemia due to capillary loss, are involved in the pathogenesis of DR^3^, but how these abnormalities affect visual function in patients is unclear.

Retinal sensitivity (RS) is a common measure of visual function^4^. Fluorescein angiography (FA) studies have demonstrated significant reduction in RS in non-perfused areas^5,6^, suggesting that perfusion status affects RS. Unlike FA, optical coherence tomography angiography (OCTA) allows quantification of flow density (FD)^7^: several studies have shown that FD is associated with RS in DR^8,9^.

Enhanced vascular permeability can cause retinal edema, followed by retinal neurodegeneration^10^. OCTA is a non-invasive alternative to FA; however, OCTA cannot identify vascular leakage, which can occur regardless of perfusion^11^. Following a report of RS reduction in areas with greater leakage^12^, we hypothesized that the greatest RS reduction occurs in areas with reduced blood perfusion and enhanced vascular permeability. To test our hypothesis, we used microperimetry to measure RS in patients with DR. We divided the measurement points into four groups according to fluorescein leakage intensity and FD value, then compared RS reduction values among groups. Contrary to our expectations, the area with leakage showed significantly less RS reduction in the area with reduced FD. Stratified analysis by retinal thickness (RT) showed that leakage could maintain RS only in areas where RT and FD were both low. Although prolonged edema due to enhanced vascular permeability promotes impairment of visual function^13^, our results suggest that leakage may inhibit the RS reduction in the non-edematous ischemic retina in patients with DR.

## Methods

This study was conducted in accordance with the principles of the Declaration of Helsinki and approved by the Institutional Review Board of Kyushu University Hospital (28-473, UMIN 000028656); written informed consent was obtained from all healthy volunteers as well as patients prior to participation in the study.

### Participants

This cross-sectional, prospective, comparative, consecutive case series included 14 eyes of 14 patients with DR who visited the Department of Ophthalmology, Kyushu University Hospital, between February 2017 and January 2020. All patients underwent microperimetry, FA, OCT and OCTA on the same day. Given the poor fixation in patients with macular edema^14^, patients with center-involving macular edema (central macular thickness ≥ 300 μm on OCT) were excluded. Anti-vascular endothelial growth factor (VEGF) therapy (intravitreal injection of ranibizumab 0.5 mg) is administered to treat diabetic macular edema in our department. Because anti-VEGF agents influence retinal vascular permeability^15^, patients who had received anti-VEGF treatment in the previous 34 days were excluded^16^. Other exclusion criteria were: active proliferative DR (PDR), other macular diseases, glaucoma, or ocular surgery within the previous 6 months. Six healthy volunteers were included for determination of reference RS values.

### Microperimetry

An MP-3 microperimeter (Nidek Co. Ltd, Aichi, Japan) was used for RS measurements. The stimulus dynamic range was 0–34 dB. The background luminance was 31.4 asb, maximum luminance was 10,000 asb, and the stimulus was Goldmann size III. RS was measured in the upper or lower temporal area of the macula (a 3 × 3-mm square with 25 points/patient; see Step 1 in Figure, Supplemental Figure 1, which demonstrates FD calculation and leakage evaluation). Only reliable data (false-positive and false-negative rates < 33%) were analyzed.

Measured values were corrected (based on RS values from six healthy volunteers) because RS decreases with increasing eccentricity. The program for measurement of RS reference values is shown in Figure, Supplemental Figure 2A. RS values at 2-degree intervals were averaged and used as reference values (see Figure, Supplemental Figure 2B, which demonstrates RS values in the healthy volunteers). The difference between measured and reference values was used as the corrected value for each test location (see Figure, Supplemental Figure 2C, which demonstrates calculation of corrected values).

### FA imaging

FA was performed using a Spectralis HRA (Heidelberg Engineering, Heidelberg, Germany). Late-phase images obtained within 7 min after dye injection were used to assess the extent of vascular leakage.

### OCTA imaging

OCTA was performed as previously reported^7,17^, using a Cirrus HD-OCT model 5000 (Carl Zeiss, Dublin, CA, USA); 3 × 3-mm images of the total, superficial and deep capillary plexus layers (TCP, SCP and DCP, respectively) of the retina were acquired at the upper- or lower-temporal side of the macula, to avoid the foveal avascular zone.

### Measurement of FD and vascular leakage

The FA image was first merged with the MP-3 image, and RS locations were marked (see Steps 1 and 2 in Figure, Supplemental Figure 1, which demonstrates calculation of FD and leakage). When the MP-3 image was deleted from the merged image, the RS locations remained on the FA image (see Step 3 in Figure, Supplemental Figure 1). The OCTA image was merged with this image; a 500-µm-diameter circle was drawn around each dot for FD measurement (see Step 4 in Figure, Supplemental Figure 1). In Supplemental Figure 1, the leftmost 5 points were excluded because 500-µm-diameter circles could not be drawn at those points; the remaining 20 points were included in the analysis. FD was measured as previously reported^7,17^ (see “Analysis 1”, Step 4, in Figure, Supplemental Figure 1). In brief, OCTA images were binarized using ImageJ (National Institutes of Health, Bethesda, MD, USA). The vessel area within the circle was calculated; the value of the vessel area/circle area was used as the FD at each location. Points at which the FD of the TCP was ≤ 0.07 were regarded as the “low” group^7^. That designation allowed investigation of the effects of leakage on RS in perfused and non-perfused areas. For SCP and DCP, points below the median were regarded as the “low” group. Finally, the OCTA image was erased from the merged FA-OCTA image, and the image with the circle on the FA was created (see Step 5 in Figure, Supplemental Figure 1). Two retinal specialists (MA and YK) independently evaluated the leakage. In case of disagreement, a third observer (SN) evaluated leakage intensity. Leakage in the circle was considered “low” if its brightness was below that of the nearby vessels and “high” if its brightness was equal or greater (see “Analysis 2”, Step 5 in Figure, Supplemental Figure 1).

### OCT imaging

Central macular thickness was measured with the Cirrus HD-OCT model 5000, as was the retinal thickness (RT) between the retinal pigment epithelium and the inner limiting membrane at each RS location.

### Statistical analysis

All values are presented as means ± standard deviations. The data were analyzed using non-parametric statistics. R software version 3.6.3 (R Foundation for Statistical Computing, Vienna, Austria) was used to calculate Spearman's rank correlation coefficients and corresponding 95% confidence intervals. JMP Pro version 14 software (SAS Institute Inc., Cary, NC, USA) was used for other analyses (Steel–Dwass test or Wilcoxon rank-sum test). The smoothing curve in scatterplots of RT versus RS was obtained using locally weighted scatterplot smoothing regression. *P* values < 0.05 were considered statistically significant.

## Results

### Patients

Fourteen patients were enrolled in the study. Data from two patients were excluded because poor eye fixation prevented acquisition of analyzable OCTA images. Although 300 RS measurements were collected (25 locations × 12 patients), FD could only be measured for 207 measurements. The characteristics of the 12 patients are shown in Table 1.

**Table 1:** Characteristics of the study population.

Eyes, n	12
RS measurement points for analysis, n	207
Male sex, n (%)	10 (83%)
Age, years	61.2 ± 14.5
HbA_1c_, %	8.0 ± 1.8
logMAR	0.09 ± 0.20
CMT, μm	277 ± 25
Days since last IVR*	99 ± 100

The data are presented as the mean ± standard deviation or as the number (percentage). *Seven of 12 patients had a history of IVR. *RS* retinal sensitivity, *CMT* central macular thickness, *HbA*_*1c*_ glycated hemoglobin, *IVR* intravitreal injection of ranibizumab, *logMAR* logarithm of the minimum angle of resolution.

### Impact of vascular leakage on the correlation between FD and RS reduction

**Figure 1:**
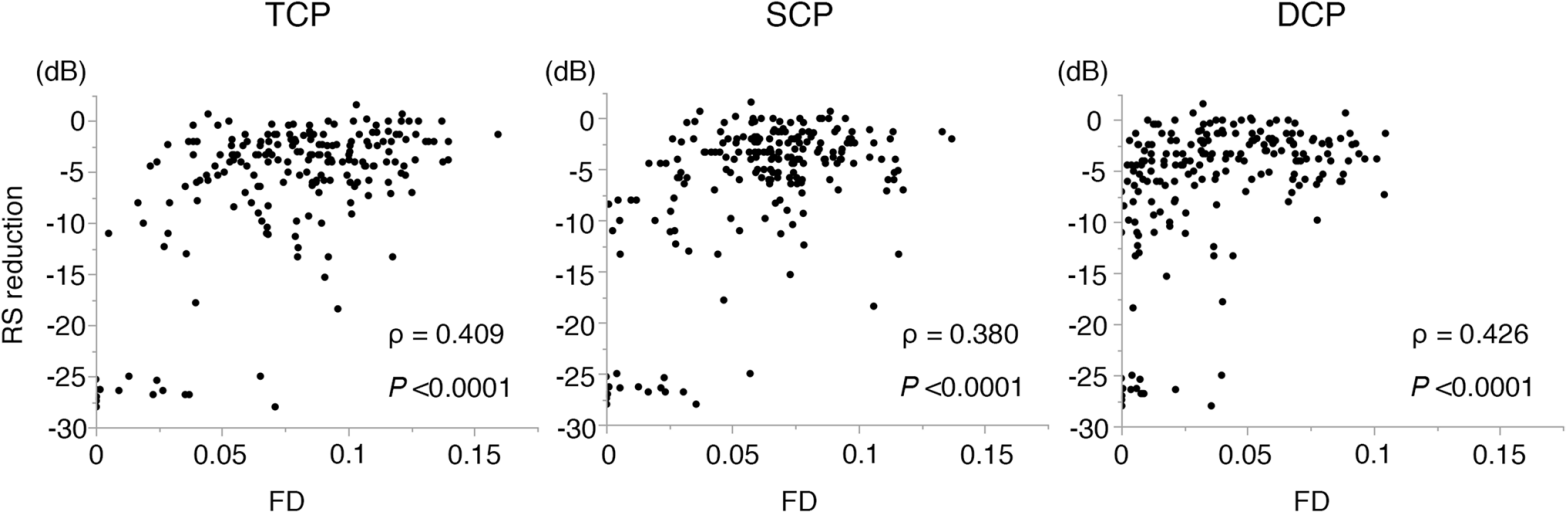
Correlations between flow density and reduction in retinal sensitivity. Scatter plots of TCP-FD, SCP-FD, or DCP-FD versus reduction in RS. Spearman's correlation coefficient (ρ) was calculated. *FD* flow density, *RS* retinal sensitivity, *TCP* total capillary plexus layer, *SCP* superficial capillary plexus layer, *DCP* deep capillary plexus layer.

A significant association between FD and RS in the diabetic macula was previously reported^18^. Similarly, FD values in the TCP, SCP and DCP were significantly correlated with RS in our study (Fig. 1). To investigate whether leakage affected this correlation, we divided all points into two groups, by their degree of leakage (“high” or “low”), and re-examined the correlation between FD and RS. Leakage did not impact the correlation in the DCP, but the correlation between FD and RS tended to be weaker in the high-leakage group than in the low-leakage group for TCP and SCP (see Figure, Supplemental Fig. 3, which demonstrates the impact of leakage on correlations between RS reduction and FD; Table 2). These results suggest that leakage could affect the FD–RS relationship in the TCP and SCP, but not the DCP.

**Table 2:** Comparison of 95% confidence interval for Spearman's rank correlation coefficient (ρ).

	High leakage (n = 109)			Low leakage (n = 98)		
	ρ	95% CI	*P* value	ρ	95% CI	*P* value
TCP-RS	0.232	0.046–0.403	0.02	0.541	0.384–0.668	< 0.0001
SCP-RS	0.304	0.123–0.466	0.001	0.449	0.275–0.595	< 0.0001
DCP-RS	0.423	0.256–0.566	< 0.0001	0.427	0.250–0.576	< 0.0001

TCP, SCP, DCP-RS: the relationship of TCP, SCP, and DCP and RS with FD. *CI* confidence interval, *DCP* deep capillary plexus layer, *SCP* superficial capillary plexus layer, *TCP* total capillary plexus layer. R: A language and environment for statistical computing. R Foundation for Statistical Computing, Vienna, Austria. URL https://www.R-project.org/.

### Classification of points according to FD and leakage

The RS locations were divided into four groups, according to the degrees of FD and leakage, for further analysis of the influence of leakage on the FD–RS relationship. We defined points with high FD and low leakage as group A, points with low FD and high leakage as group B, points with high FD and high leakage as group C, and points with low FD and high leakage as group D (see Table 3).

**Table 3:** Classification of retinal sensitivity measurement points (n = 207) by level of flow density and leakage.

**Classification method**		FD	
		High	Low
Leakage	Low	Group A	Group B
	High	Group C	Group D

**TCP**		FD	
		High	Low
Leakage	Low	62	36
	High	62	47

**SCP**		FD	
		High	Low
Leakage	Low	46	52
	High	57	52

**DCP**		FD	
		High	Low
Leakage	Low	57	41
	High	46	63

*FD* flow density, *TCP* total capillary plexus layer, *SCP* superficial capillary plexus layer, *DCP* deep capillary plexus layer.

### RS reductions among the four groups

**Figure 2:**
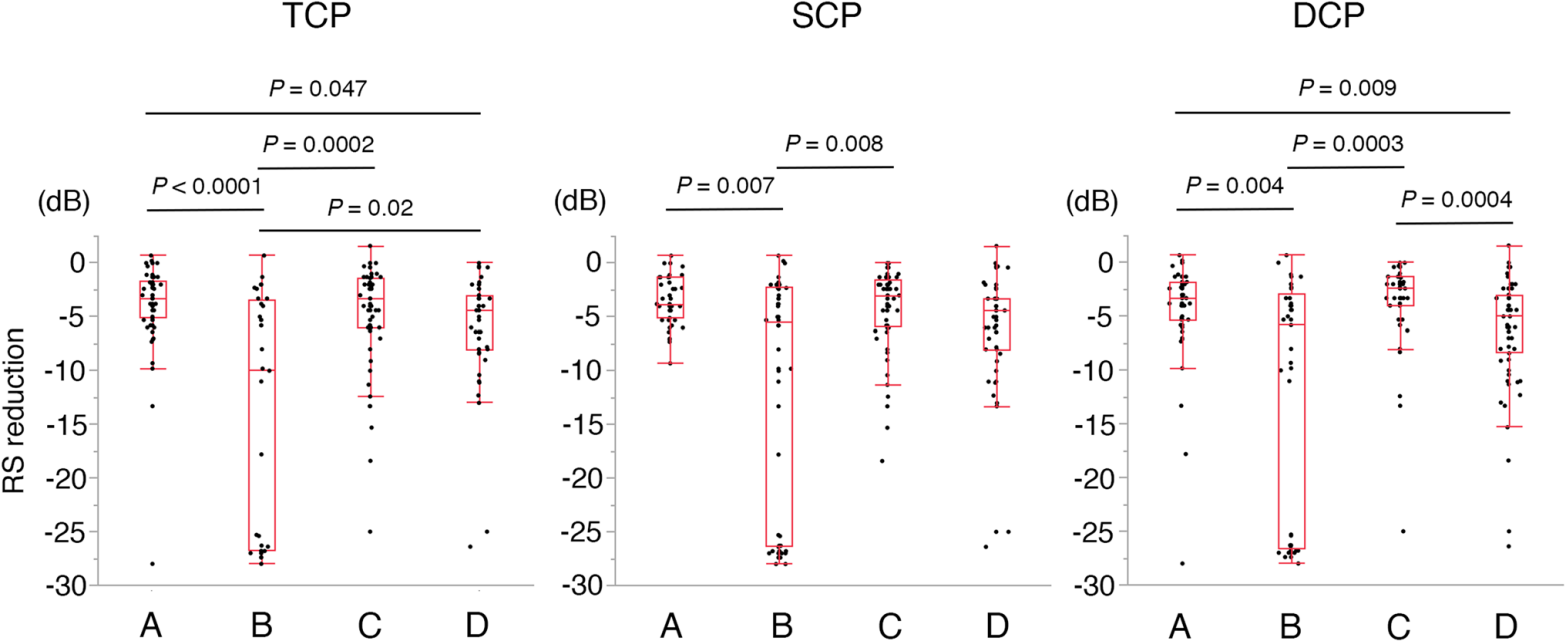
Comparison of reductions in retinal sensitivity among the four groups. All RS measurement points were classified into four groups according to FD and vascular leakage. Group A: high FD and low leakage, Group B: low FD and low leakage, Group C: high FD and high leakage, Group D: high FD and high leakage. The Steel–Dwass test was used for statistical analyses. *FD* flow density, *RS* retinal sensitivity.

Figure 2 compares RS reductions among the groups. For TCP, the RS reduction values were − 3.9 ± 4.1 dB, − 14.2 ± 11.2 dB, − 4.6 ± 4.8 dB, and − 6.0 ± 5.4 dB in groups A, B, C, and D, respectively. At locations with low leakage, RS reduction was significantly greater in the low-FD group than in the high-FD group (group A vs. group B, *P* < 0.0001). However, in groups with high leakage, there was no significant difference in RS reduction between points with low FD and high FD (group C vs. group D, *P* = 0.21). At locations where FD was high, the degree of leakage did not influence RS reduction (group A vs. group C, *P* = 0.95). Conversely, at locations where FD was low, RS reduction was significantly smaller in the high-leakage group than in the low-leakage group (group B vs. group D, *P* = 0.02).

For SCP, the RS reductions were − 3.4 ± 2.3 dB, − 11.4 ± 10.9 dB, − 4.3 ± 4.0 dB, and − 6.2 ± 6.0 dB in groups A, B, C, and D, respectively. RS reduction was significantly greater in the low-FD group with low leakage than in the high-FD group with low leakage (group A vs. group B, *P* = 0.007). For DCP, the RS reductions were − 4.2 ± 4.5 dB, − 12.5 ± 11.3 dB, − 3.6 ± 4.3 dB, and − 6.4 ± 5.3 dB in groups A, B, C and D, respectively. RS reduction in DCP was significantly greater in the low-FD group at locations with either low leakage (*P* = 0.004) or high leakage (*P* = 0.0004). At locations where FD was high, the degree of leakage did not influence RS reduction (group A vs. group C, *P* = 0.51). Likewise, where FD was low, the degree of leakage did not influence RS reduction (group B vs. group D, *P* = 0.33).

### Effect of RT on RS reduction

**Figure 3:**
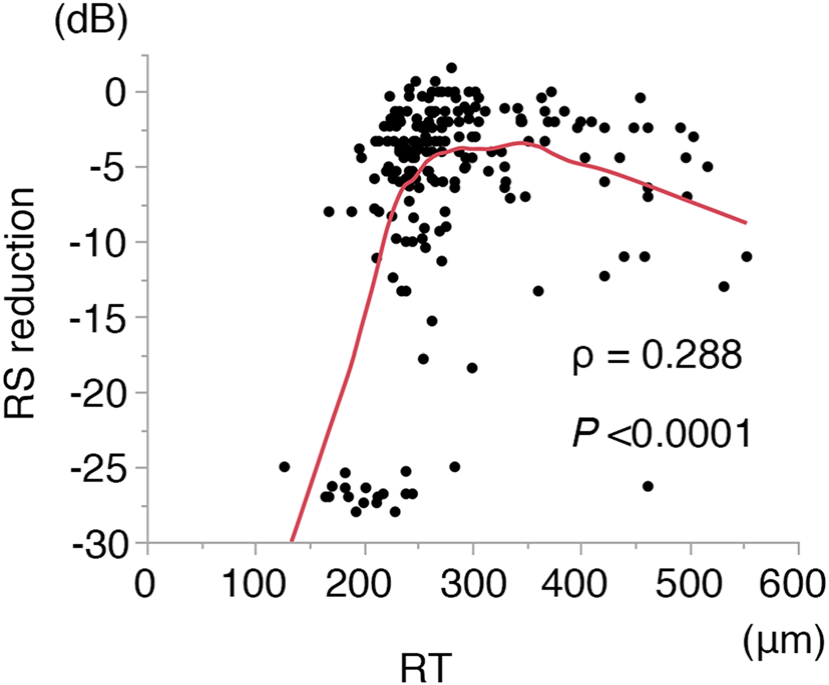
Correlations between retinal thickness and reduction in retinal sensitivity. Scatter plots of RT versus reduction in RS. Spearman's correlation coefficient (ρ) was calculated. Red line shows locally weighted scatterplot smoothing curve. *RT* retinal thickness, *RS* retinal sensitivity.

We started by measuring RT at all 207 points. There were no points with subretinal fluid that could affect RS in these patients^19^. Scatterplots of RT versus RS (Fig. 3) show a weak correlation between RT and RS. Consistent with previous reports^20,21^, RS reduction in our patients tended to decrease with increasing RT, until the RT reached approximately 300 μm; RS reduction tended to increase with increasing RT when the RT exceeded approximately 300 μm. Considering the effect of RT on RS, we performed a stratified analysis.

### Possible suppression of RS reduction by vascular leakage

All measurements were divided into two groups: locations where RT values exceeded the median (261 μm) and those where RTs were less than or equal to the median. Locations were then subdivided according to high or low FD. The results of analyses using high-RT and low-RT locations are shown in Tables 4 and 5, respectively. In the high-RT group, vascular leakage did not influence RS reduction, regardless of whether FD was high or low (Table 4). In the low-RT group, RS reduction was unaffected by vascular leakage at points with high FD but was affected at points with low FD (Table 5). RS reduction was significantly smaller at points with high leakage than at points with low leakage in the TCP, SCP and DCP subgroups (Table 5).

**Table 4:** Comparison of retinal sensitivity at each flow density between the high and low leakage groups with high retinal thickness (> 261 μm).

Leakage	High	Low	*P* value*
**High FD in TCP**			
n	44	32	–
RS reduction, dB	− 3.9 ± 4.1	− 3.2 ± 2.5	0.87
**Low FD in TCP**			
n	20	7	–
RS reduction, dB	− 6.3 ± 6.0	− 7.0 ± 9.1	0.76
**High FD in SCP**			
n	42	27	–
RS reduction, dB	− 4.0 ± 4.2	− 3.3 ± 2.6	0.89
**Low FD in SCP**			
n	22	12	–
RS reduction, dB	− 5.8 ± 5.9	− 5.1 ± 7.2	0.29
**High FD in DCP**			
n	26	26	–
RS reduction, dB	− 3.4 ± 5.2	− 3.0 ± 2.3	0.44
**Low FD in DCP**			
n	38	13	–
RS reduction, dB	− 5.4 ± 4.5	− 5.6 ± 7.0	0.40

*Wilcoxon rank-sum test. *DCP* deep capillary plexus layer, *FD* flow density, *RS* retinal sensitivity, *SCP* superficial capillary plexus layer, *TCP* total capillary plexus layer.

**Table 5:** Comparison of retinal sensitivity at each flow density between the high and low leakage groups with low retinal thickness (≤ 261 μm).

Leakage	High	Low	*P* value*
**High FD in TCP**			
n	18	30	–
RS reduction, dB	− 6.4 ± 5.9	− 4.6 ± 5.2	0.19
**Low FD in TCP**			
n	27	29	–
RS reduction, dB	− 5.8 ± 5.0	− 15.9 ± 11.1	0.001
**High FD in SCP**			
n	15	19	–
RS reduction, dB	− 5.0 ± 3.2	− 3.5 ± 1.7	0.25
**Low FD in SCP**			
n	30	40	–
RS reduction, dB	− 6.6 ± 6.1	− 13.3 ± 11.1	0.04
**High FD in DCP**			
n	20	31	–
RS reduction, dB	− 3.8 ± 2.9	− 5.2 ± 5.8	0.47
**Low FD on DCP**			
n	25	28	–
RS reduction, dB	− 7.8 ± 6.2	− 15.7 ± 11.5	0.046

*Wilcoxon rank-sum test. *DCP* deep capillary plexus layer, *FD* flow density, *RS* retinal sensitivity, *SCP* superficial capillary plexus layer, *TCP* total capillary plexus layer.

### Comparisons of RT among groups

RT values in the four groups, classified by FD and leakage levels, are shown in Figure, Supplemental Figure 4. For TCP, the RT values were 263 ± 34 μm, 245 ± 68 μm, 312 ± 80 μm, and 301 ± 102 μm in groups A, B, C, and D, respectively. For SCP, the RT values were 268 ± 34 μm, 247 ± 59 μm, 318 ± 79 μm, and 296 ± 100 μm in groups A, B, C, and D, respectively. For DCP, the RS values were 259 ± 34 μm, 253 ± 66 μm, 287 ± 56 μm, and 323 ± 106 μm in groups A, B, C, and D, respectively. For TCP and SCP, the RT values were significantly greater in group C than in group A (*P* = 0.02 and *P* = 0.02, respectively). There were no significant differences between groups C and D in any layers. For DCP, the RT was significantly greater in group D than in group B (*P* = 0.005). For TCP and SCP, the RTs were significantly greater in group C than in group A (*P* = 0.02 and *P* = 0.003, respectively).

## Discussion

Our data show that RS reduction was inhibited under certain conditions, in patients with DR, even with reduced vascular flow. In areas without significant edema, RS was maintained in areas with vascular leakage, compared with faint or no leakage, despite capillary dropout.

In DR, vascular permeability is enhanced owing to disruption of the inner blood-retinal barrier (BRB) because of ischemia, inflammation and oxidative stress^22–24^. Disruption of BRB function leads to accumulation of retinal fluid and influx of harmful substances into the retina, which lead to irreversible degeneration of neurons, including photoreceptors^10^. VEGF plays a major role in BRB disruption^23,25,26^. Several clinical trials have demonstrated significant improvement in visual acuity with continued anti-VEGF therapy in patients with DR^13,27,28^, so vascular hyperpermeability is generally considered an “unfavorable” phenomenon, which should be suppressed.

Our study did not show that leakage negatively influenced RS, which raises the question of why leaky retina retained more RS, than did less-leaky retina, in low-FD areas. Where it has not completely lost RS, the retina may develop vascular leakage though VEGF secretion. To our knowledge, there have been no reports that leakage positively influences retinal function. Elucidating the multifaceted role of vascular leakage may help clarify the pathogenesis of DR, including the pattern of neurodegeneration.

In the context of our results, we cannot exclude the possibility that anti-VEGF therapy caused side effects on the neuronal retina under certain circumstances^29^. Because reduced RS around the fovea can lead to poor vision quality, other therapies might be necessary, such as steroid treatment for patients with non-edematous ischemic diabetic retina^2^. However, further studies are needed to identify the patients in whom anti-VEGF therapy should be cautiously administered.

As previously reported^18^, there was a correlation between FD decline and RS reduction in DR in our study. Because RS decreases in areas with greater vascular leakage^12^, we investigated whether leakage affected the relationship between FD and RS. In the TCP and SCP, the strength of the correlation changed depending on the extent of leakage, suggesting that leakage influences the correlation between FD and RS. We did not observe a significant impact of leakage on the correlation in the DCP. Spaide et al. showed that FA does not image the DCP^30^; thus, DCP leakage may not be completely detected by FA.

As leakage altered the correlation between FD and RS, we speculated that leakage in non-perfused areas might lead to the greatest RS reduction. Therefore, we categorized all RS locations into four groups, based on FD and leakage. At points where leakage was low, RS decreased when FD decreased (group A vs. group B; *P* < 0.0001, *P* < 0.007 and *P* < 0.004 in TCP, SCP and DCP, respectively), as previously reported^9,16^. However, contrary to expectations, at points with high leakage, FD only significantly affected RS in the DCP (group C vs. group D; *P* < 0.0004). Previous reports have shown that reduced blood flow in the DCP contributes to disruption of the outer retinal layer and reduction in RS^31,32^. Reduced perfusion in the deep layer may be more likely to affect RS. Leakage did not lead to RS reduction at locations where FD values were maintained (group A vs. group C). Notably, at low-FD points in the TCP, leakage significantly inhibited the reduction in RS (group B vs. group D; *P* = 0.02). These results indicated that our hypothesis was incorrect and suggested that leakage may not be uniformly "unfavorable."

We subsequently investigated whether leakage could suppress RS reduction. RT and RS are not linearly correlated because edema causes loss of visual function^33^. Analysis of our high-RT group revealed no significant difference in RS reductions between areas of high and low leakage. However, analysis of the low-RT group revealed that leakage significantly inhibited the reduction in RS. These results suggest that the retina, which undergoes atrophy due to DR, can maintain RS despite leakage. We could not show that vascular leakage adversely affected RS.

A comparison of RT values between groups is shown in Figure, Supplemental Figure 4. In all analyses, group B had the lowest RT. As shown in Fig. 2, group B showed the greatest reduction in RS; however, RS reduction exhibited polarization. Based on these results, we considered the following hypotheses regarding RS reduction (see Figure, Supplemental Figure 5): 1) Edema is formed at points with normal FD and high leakage (group A to C). Then, circulatory disturbances by edema exacerbate ischemia (group C to D) and eventually promote retinal atrophy (group D to B). 2) After ischemia reduces FD (group A to B), leakage increases (group B to D) and edema occurs. Cellular damage and further edema-related circulatory disturbances lead to retinal atrophy (groups D to B). 3) If the degree of ischemia is severe, the retina may atrophy without enhanced vascular permeability or edema (group A to B).

The lack of consideration of the time since DR onset was a limitation of this study. Leakage may not have had an "unfavorable" effect on RS because DR duration was not considered. Longitudinal studies are needed to follow patients over time and elucidate pathways that lead to neurodegeneration. Because patients with center-involved macular edema or PDR were excluded from our study population, the associations of vascular permeability with FD, RT, and RS observed in this study may not be generalizable to all patients with DR. Furthermore, our study included a small sample of patients, although the number of RS measurements was high.

In conclusion, vascular leakage in non-perfused areas suppressed the reduction in RS in eyes with DR. Our results suggest that vascular leakage has a negative impact in terms of damaging the retina; it also has a positive impact in terms of preserving ischemic retinal function.

## Supplementary Information

Supplementary Information 1.
