# Publisher Correction: Diabetic vascular hyperpermeability: optical coherence tomography angiography and functional loss assessments of relationships among retinal vasculature changes

**DOI:** 10.1038/s41598-021-91789-w

**Published:** 2021-06-07

**Authors:** Mitsuru Arima, Shintaro Nakao, Yoshihiro Kaizu, Iori Wada, Muneo Yamaguchi, Kohta Fujiwara, Masato Akiyama, Alan W. Stitt, Koh‑Hei Sonoda

**Affiliations:** 1 Department of Ophthalmology, Graduate School of Medical Sciences, Kyushu University, Fukuoka, Japan; 2 Department of Ophthalmology, National Kyushu Medical Center, 1‑8‑1, Jigyo‑hama, Chuo‑ku, Fukuoka, 8108563 Japan; 3 Department of Ophthalmology, Fukuoka University Chikushi Hospital, Fukuoka, Japan; 4 Centre for Experimental Medicine, Queen’s University Belfast, Belfast, Northern Ireland

Correction to: *Scientific Reports* 10.1038/s41598-021-83334-6, published online 18 February 2021

The original version of this Article contained an error in Table 3, where a heading in the subcategory “Classification method” was incorrect. The correct and incorrect values appear below.

Incorrect:

**Table:** 

Classification method		FD	
		High	Low
90 pt	Low	Group A	Group B
	High	Group C	Group D

Correct:

**Table:** 

Classification method		FD	
		High	Low
Leakage	Low	Group A	Group B
	High	Group C	Group D

This error has now been corrected in the PDF version; the HTML version was correct from the time of publication.

