# Supplementary material for: Diabetic vascular hyperpermeability: optical coherence tomography angiography and functional loss assessments of relationships among retinal vasculature changes
Source: Sci Rep. 2021 Feb 18;11:4185. doi: 10.1038/s41598-021-83334-6 (PMC7892857; doi:10.1038/s41598-021-83334-6)
Supplement: Supplementary file 1 — Supplementary Information 1. [file 41598_2021_83334_MOESM1_ESM.pdf]

Diabetic vascular hyperpermeability: optical coherence tomography angiography and functional loss assessments of relationships among retinal vasculature changes

Mitsuru Arima[1], Shintaro Nakao[1,2], Yoshihiro Kaizu[1], Iori Wada[1], Muneo Yamaguchi[1,3], Kohta Fujiwara[1], Masato Akiyama[1], Alan W. Stitt[4] & Koh-Hei Sonoda[1]

1.Department of Ophthalmology, Graduate School of Medical Sciences, Kyushu University, Fukuoka, Japan
2.Department of Ophthalmology, National Kyushu Medical Center, Fukuoka, Japan
3.Department of Ophthalmology, Fukuoka University Chikushi Hospital, Fukuoka, Japan
4.Centre for Experimental Medicine, Queen's University Belfast, Belfast, Northern Ireland

Corresponding author: Dr. Shintaro Nakao, MD, PhD.
Department of Ophthalmology, National Kyushu Medical Center, Fukuoka, Japan
1-8-1, Jigyo-hama, Chuo-ku, Fukuoka 8108563, Japan
Tel: +81-92-852-0700, Fax: +81-92-847-8802
E-mail: snakao@med.kyushu-u.ac.jp

Supplementary Figure 1

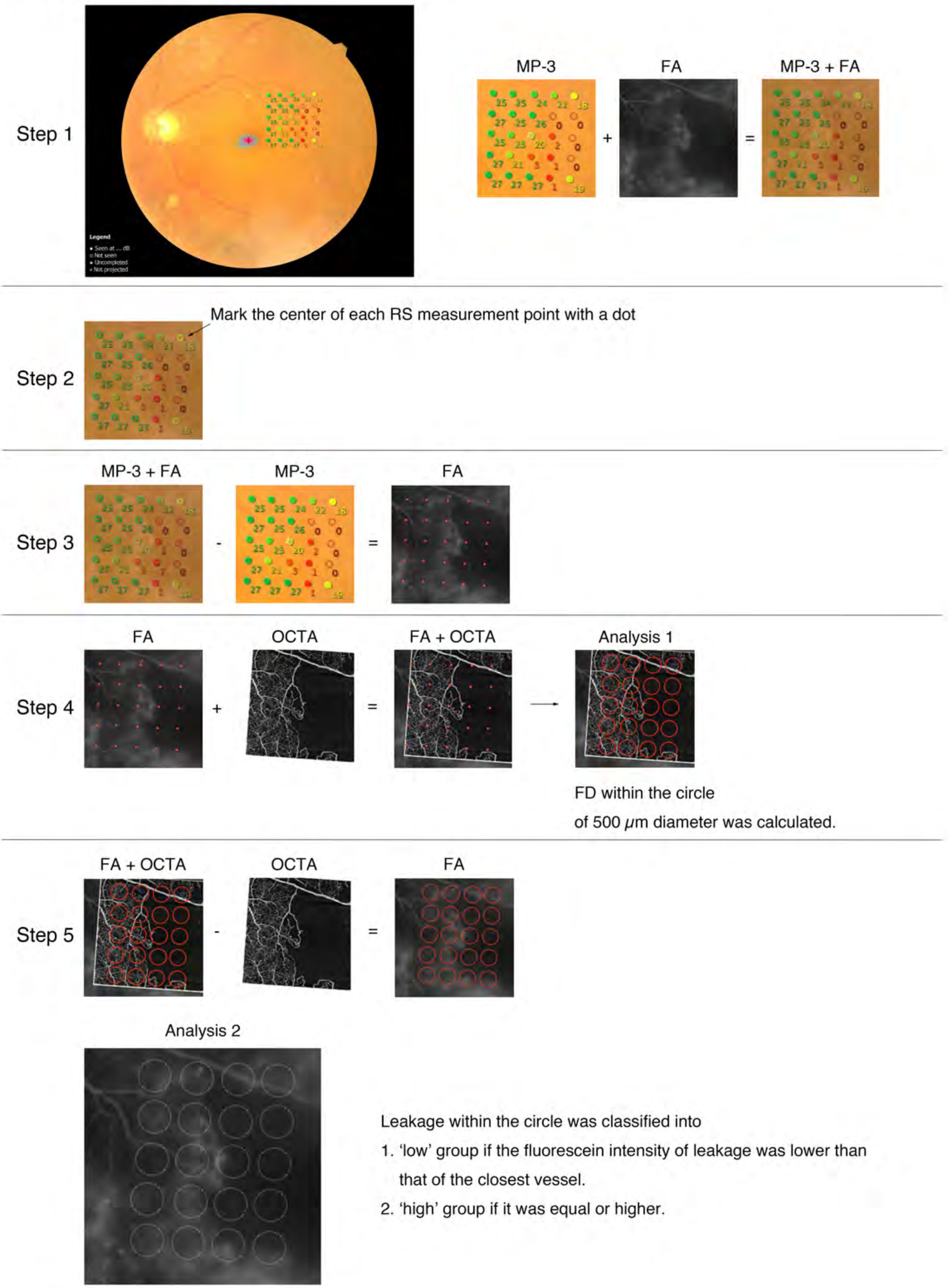

Supplementary Figure 2

A

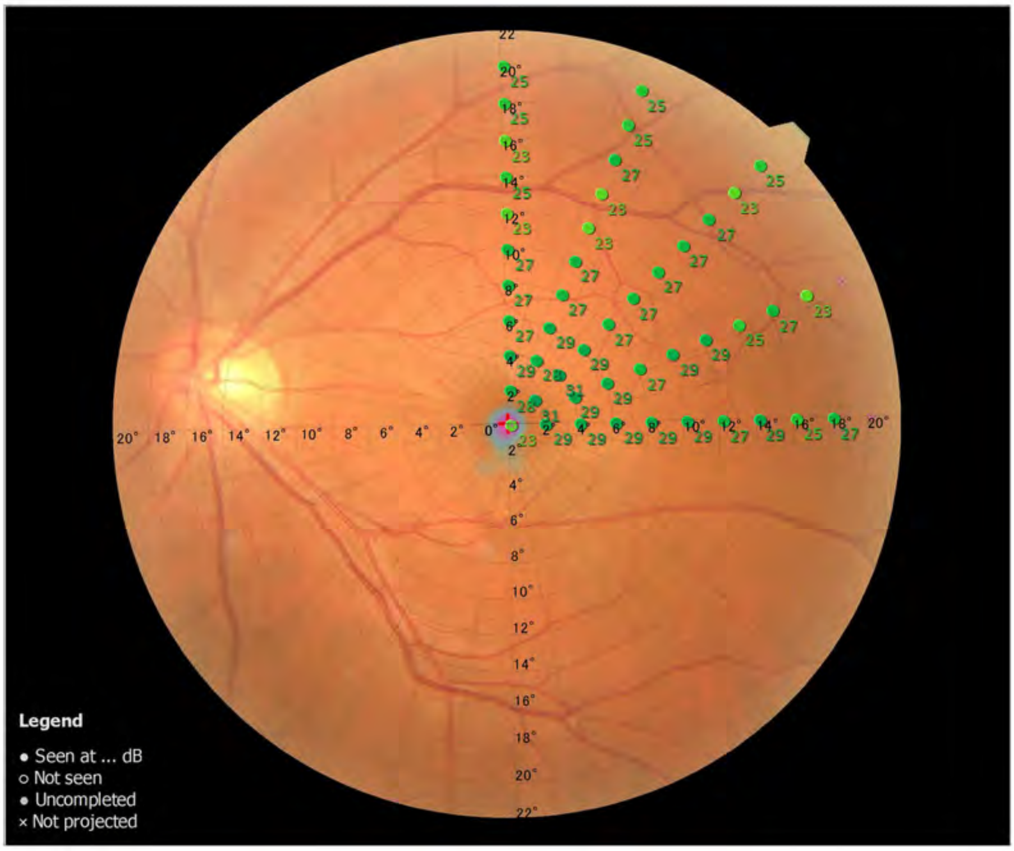

B

	2°	4°	6°	8°	10°	12°	14°	16°	18°
No. 1 (dB)	29.3	29.2	28.6	27.4	27.4	26.2	25.8	25.8	24.6
No. 2 (dB)	31.3	29.8	29	28.4	27.8	27.8	28.2	27.8	26.2
No. 3 (dB)	30.7	30	28.8	29.4	27.8	27	27.2	28.2	27
No. 4 (dB)	28.3	27.4	26.8	27	27.6	27.4	25.8	25	23.8
No. 5 (dB)	30	27.8	26.6	27.4	25.8	26.6	24.6	25.8	23.8
No. 6 (dB)	30.3	28.6	28.6	28.2	27.8	27	26.2	28	26.6
Reference value (mean, dB)	30	28.8	28.1	28.0	27.4	27	26.3	26.8	25.3

C

Assign an angle to each measurement point

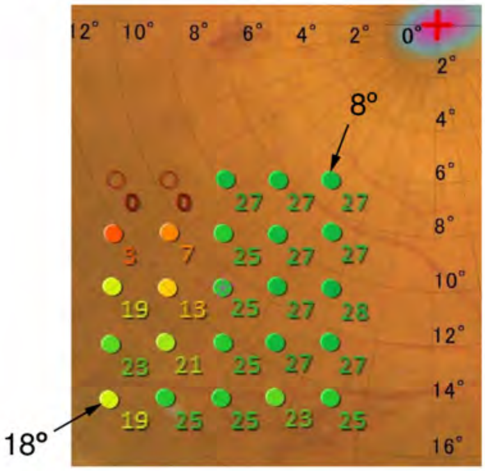

Measured value

0	0	27	27	27
3	7	25	27	27
19	13	25	27	28
23	21	25	27	27
19	25	25	23	25

→

Corrected value

-26.3	-27.0	-0.4	-1.0	-1.0
-23.3	-20.0	-2.0	-0.4	-1.0
-7.8	-13.3	-2.0	0	0.6
-3.8	-5.8	-1.3	0.7	0
-6.3	-0.3	-1.8	-3.8	-1.3

Subtract the reference value

Supplementary Figure 3

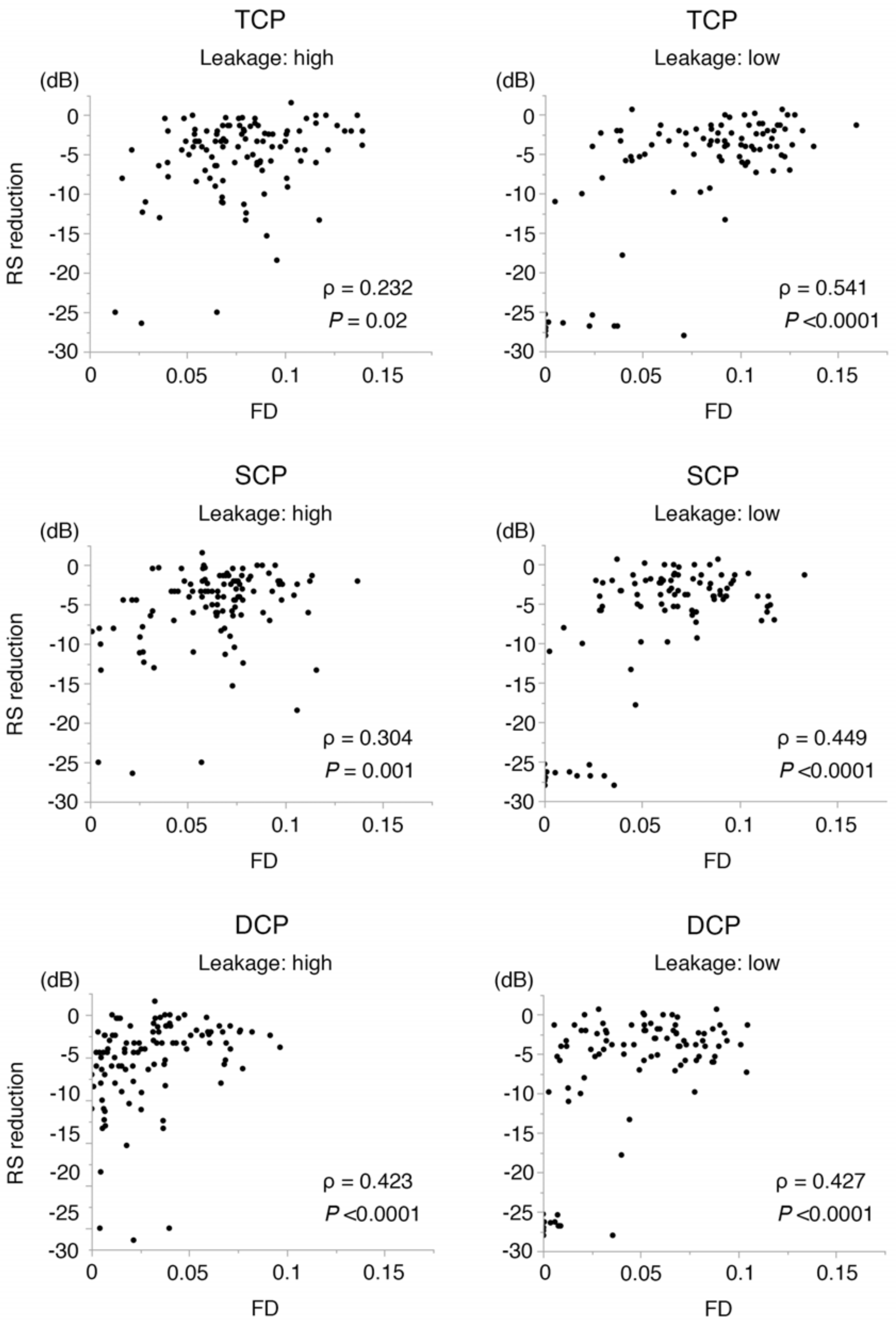

Supplementary Figure 4

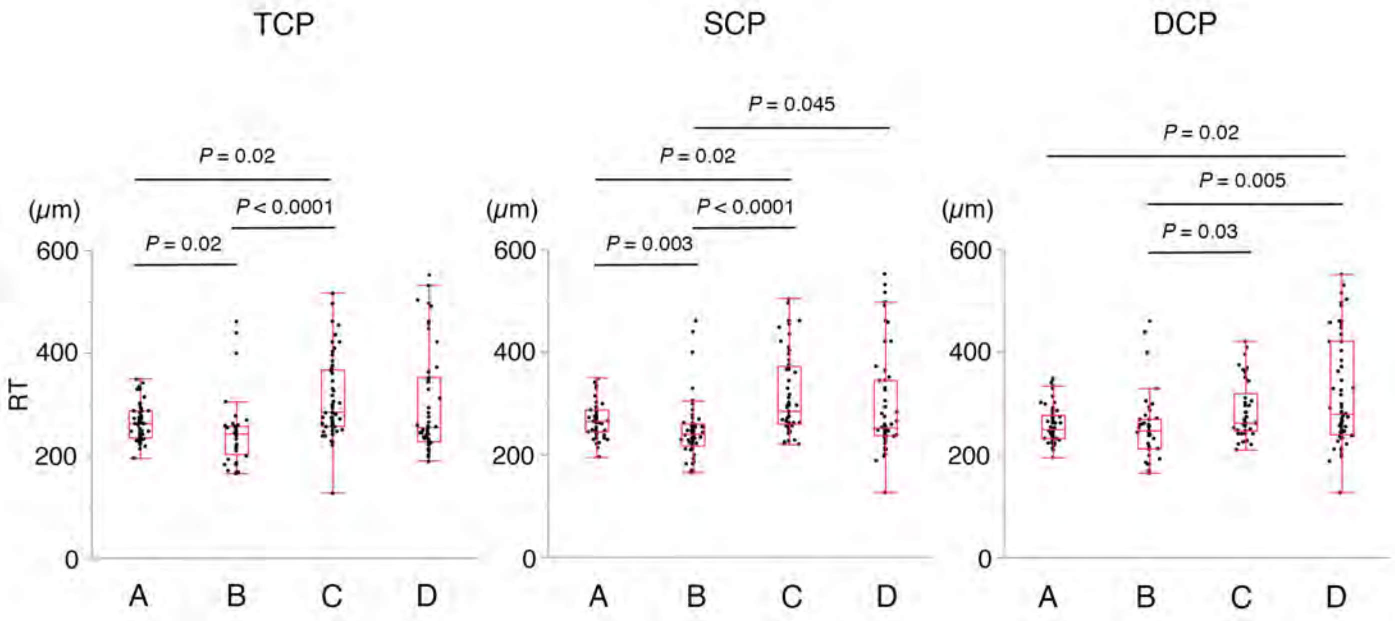

Supplementary Figure 5

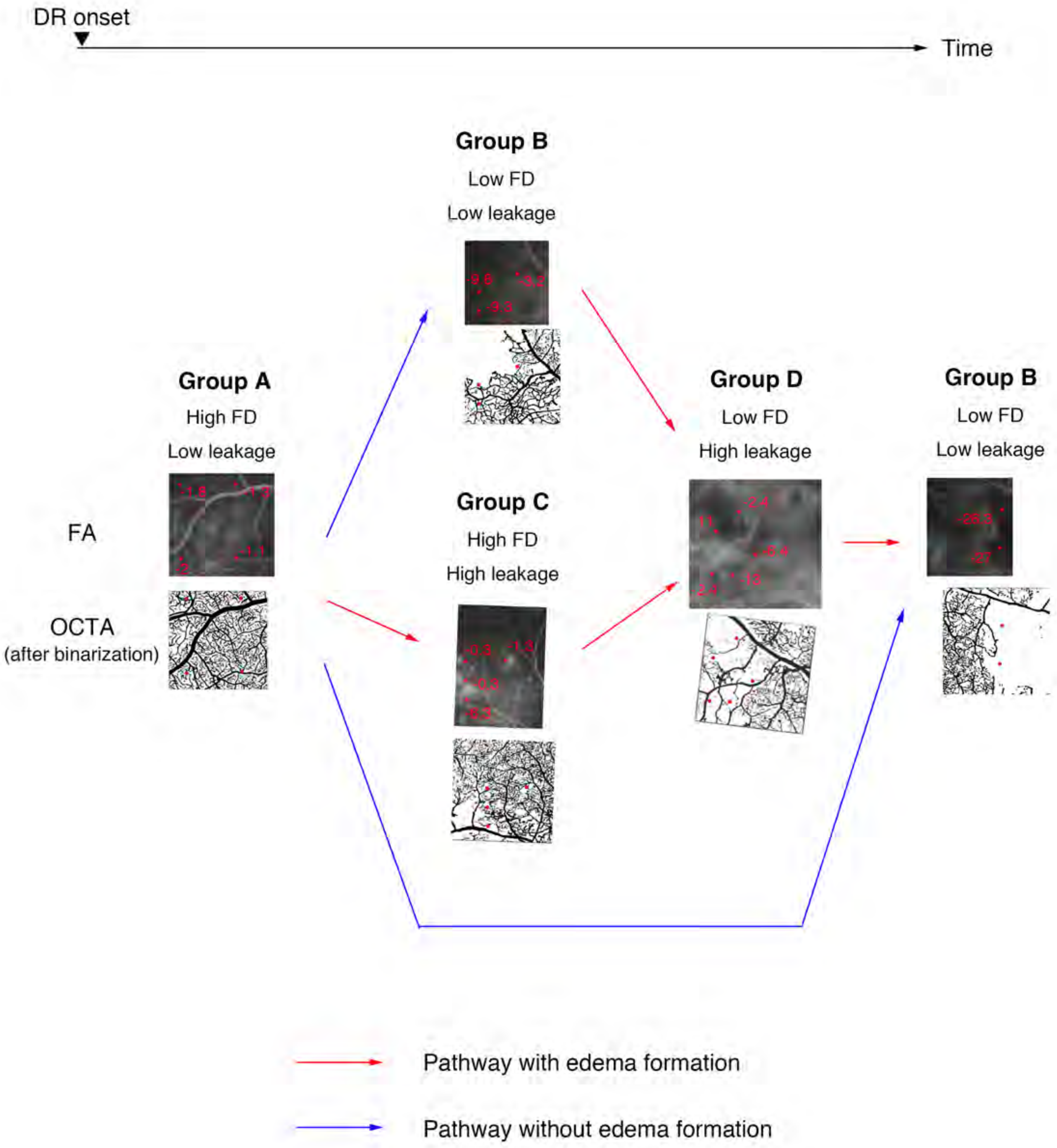

Supplemental figure legends

Supplemental Figure 1. Procedures used to calculate flow density and evaluate leakage. FD values in a 500-μm-diameter circle around RS measurement points were calculated (Analysis 1); leakage in the circle was classified as high or low (Analysis 2). MP-3 = microperimeter, FA = fluorescein angiography, FD = flow density, OCTA = optical coherence tomography angiography, RS = retinal sensitivity

Supplemental Figure 2. Retinal sensitivity measurement programs used in healthy volunteers and correction methods used for retinal sensitivity in patients with diabetic retinopathy. A: RS measurement program used to create reference values. Three RS points were measured at the 2° position; five RS points were measured at intervals of 2°, from 4° to 20°. B: RS measurement values in six healthy volunteers. A reference value for 20° could not be generated because RS could not be measured for all five points at 20°. C: Reference value of the corresponding angle was subtracted from the measured value to derive the corrected value. RS = retinal sensitivity

Supplemental Figure 3. Impacts of leakage on correlations of reduction in retinal sensitivity with flow density in the TCP, SCP, and DCP. Spearman's correlation coefficient (ρ) was calculated. FD = flow density, RS = retinal sensitivity, TCP = total capillary plexus layer, SCP = superficial capillary plexus layer, DCP = deep capillary plexus layer. R Core Team (2020). R: A language and environment for statistical computing. R Foundation for Statistical Computing, Vienna, Austria. URL https://www.R-project.org/.

Supplemental Figure 4. Comparison of retinal thicknesses among the four groups. All RS measurement points were classified into four groups according to FD and vascular leakage. Group A: high FD and low leakage, Group B: low FD and low leakage, Group C: high FD and high leakage, Group D: high FD and high leakage. The Steel-Dwass test was used for statistical analyses. RT = retinal thickness, TCP = total capillary plexus layer, SCP = superficial capillary plexus layer, DCP = deep capillary plexus layer.

Supplemental Figure 5. Expected mechanism underlying RS reduction in patients with diabetic retinopathy, in accordance with changes in FD and vascular leakage. FA (upper) and OCTA images (lower) for each group are shown. Red letters on FA images indicate RS reduction values. The results suggest the presence of at least three pathways, two with edema formation (A→B→D→B and A→C→D→B), and one

without edema formation (A→B). FA = fluorescein angiography, FD = flow density, OCTA = optical coherence tomography angiography, RS = retinal sensitivity.
